# Combined use of Amplified Fragment Length Polymorphism and IS*6110*-RFLP in fingerprinting clinical isolates of *Mycobacterium tuberculosis *from Kerala, South India

**DOI:** 10.1186/1471-2334-7-86

**Published:** 2007-07-28

**Authors:** Manju Y Krishnan, Indulakshmi Radhakrishnan, Biljo V Joseph, Madhavi Latha GK, Ajay Kumar R, Sathish Mundayoor

**Affiliations:** 1 Rajiv Gandhi Centre for Biotechnology, Thiruvananthapuram- 695 014, Kerala, India

## Abstract

**Background:**

DNA fingerprinting by IS*6110*-RFLP has shown a high incidence of *Mycobacterium tuberculosis *isolates having no and low copies of the insertion sequence in Kerala, South India. Amplified Fragment Length Polymorphism (AFLP) would scan the entire genome rather than a few repetitive elements, we thought that this technique would help us in differentiating the large reservoir of isolates from an endemic region. Here we evaluate the ability of Amplified Fragment Length Polymorphism (AFLP) to type clinical isolates.

**Methods:**

Fifty clinical isolates of *M. tuberculosis *were analysed by conventional radioactive AFLP and IS*6110- *RFLP. *M. bovis*, *M. bovis *BCG and two non tuberculous mycobacteria were also analysed to see species specific differences generated by AFLP. Cluster analysis was performed using the AFLP profile that showed the maximum polymorphism within *M. tuberculosis *and this was compared to the number of copies of IS*6110 *insertions.

**Results:**

For AFLP, out of ten primer pairs tested, the EO/MC pair generated maximum polymorphism among the clinical isolates of *M. tuberculosis*. The similarity between the isolates ranged between 88 and 99.5%. Majority (nearly 85%) of the 'low copy' IS*6110 *isolates clustered together, while the rest clustered irrespective of the copy numbers. AFLP could show rare differences between isolates of *M. tuberculosis*, *M. bovis *and *M. bovis *BCG. The AFLP profiles for non-tuberculous mycobacteria were highly different from those of *M. tuberculosis*.

**Conclusion:**

Polymorphism generated by AFLP within the *M. tuberculosis *species is limited and hence AFLP alone seems to have limited use in fingerprinting the isolates in Kerala. The combined use of AFLP and IS*6110*-RFLP showed relatively better differentiation of 'high copy' IS*6110 *isolates, but failed to differentiate the 'low copy' isolates. However, the technique may be efficient in inter-species differentiation, and hence potentially useful in identifying and developing species- specific markers.

## Background

Tuberculosis claims about 500, 000 deaths annually in India and the disease is endemic in Kerala, a small state in the southwest [1]. DNA fingerprinting by RFLP-hybridization based on the insertion sequence IS*6110 *[2] showed a high prevalence of 'no copy' and single copy isolates of *Mycobacterium tuberculosis *in Kerala [3]. This, and other studies point out that IS*6110*-based methods have limited use in regions where there is a high incidence of strains carrying few copies of IS*6110 *[4,5]. Spoligotyping, the next widely used fingerprinting method for *M. tuberculosis *complex organisms can efficiently differentiate 'low copy' (5 or fewer copies of IS*6110*) strains but again is not as efficient as RFLP for 'high copy' (6 or more copies of IS*6110*) strains [6,7]. Fingerprinting techniques such as Random Amplification of Polymorphic DNA (RAPD) and Amplified Fragment Length Polymorphism (AFLP) would scan the entire genome rather than a few repetitive elements, and since AFLP can be applied to any genome irrespective of its complexity [8], we thought that this technique would help us in differentiating the large reservoir of isolates from an endemic region. AFLP has been used for strain typing within different bacterial genera [9] and has also been evaluated in inter- and intra-species typing of mycobacteria [10-13]. In the present study, we analyzed clinical isolates of *M. tuberculosis *from Kerala using AFLP and looked at the ability of the technique to further differentiate isolates typed by IS*6110*-RFLP. The ability of AFLP to provide inter-species differentiation was used to prove the validity of the methodology adopted by us.

## Methods

### Clinical isolates and laboratory strains

Fifty clinical isolates of *Mycobacterium tuberculosis*, isolated from TB patients and identified based on their growth characteristics, colony morphology and biochemical tests [14] were used in this study. The Laboratory strains of mycobacteria were *M. tuberculosis *H37Rv, H37Ra, *M. bovis *and *M.bovis *BCG. Two atypical mycobacteria obtained from clinical samples were also included in the study. Using INNO-LiPA Mycobacteria (LiPA; Innogenetics, Zwijnaarde, Belgium), one was identified as *M. chelonae*, while the other could be identified to genus level only.

The isolates used in this investigation were collected by our laboratory as a part of a study to screen for anti-TB drug sensitivity and became part of our local repository. Since this was a retrospective study on the isolates already available with us, it was not put up for any clearance to an ethical committee.

### Genomic DNA

Genomic DNA was isolated from mycobacteria using CTAB method as reported earlier [2].

### AFLP analysis

AFLP adapters and primers [8] were custom synthesized by Sigma Genosys (Table 1). AFLP analysis was carried out as described by Vos *et al *[8]. Briefly, about 200 ng genomic DNA from each strain/isolate was digested with *Eco*R I and *Mse *I (New England Biolabs, Beverly, MA). Each sample of digested DNA was ligated to *Eco*R I and *Mse *I adapters (5 pmol and 50 pmol respectively). The ligated mixture was diluted to 1:10 ratio in 1X TE buffer and used as template for PCR. Just before the PCR, *Eco*R I primer was labeled with γ-^32^P (3000 Ci/mmol ATP), using T4 polynucleotide kinase (NEB). Five μl template DNA, 30 ng *Mse *I primer, 5 ng labeled *Eco*R I primer, were used for each 20 μl PCR reaction. PCR was carried out in an iCycler (Bio-Rad Labs, Hercules, CA). As described by Vos *et al *(8), the first cycle was carried out at 94°C for 30 sec, 65°C for 30 sec and 72°C for 60 sec. The annealing temperature was lowered each cycle by 0.7°C during the next 12 cycles (touch down phase of 13 cycles) and finally 23 cycles were carried out at 94°C for 30 sec, 56°C for 30 sec and 72°C for 60 sec.

**Table 1:** Sequences of AFLP adapters and primers(Vos et al., 1995).

AFLP adapter (*Eco*R I) oligo 1 : 5'-CTC GTA GAC TGC GTA CC-3'
AFLP adapter (*Eco*R I) oligo 2 : 5'-AAT TGG TAC GCA GTC TAC-3'
AFLP adapter (*Mse *I) oligo 1 : 5'-GAC GAT GAG TCC TGA G-3'
AFLP adapter (*Mse *I) oligo 2 : 5'-TAC TCA GGA CTC AT-3'
Primer, EO (non selective)^a ^: 5'-GAC TGC GTA CCA ATT C-3'
Primer, MO (non selective)^a ^: 5'-GAT GAG TCC TGA GTA A-3'

^a ^Selective primers were synthesized with an extra nucleotide-A, T, G or C at the 3' end of the non-selective primer sequence and were designated as EA, MT etc. accordingly.

The PCR products were analyzed by urea-PAGE. Gel images were visualized in a Phosphor imager (Personal Molecular Imager FX, Bio-Rad, Hercules, CA).

### IS*6110*-RFLP

This was performed according to the standard protocol [2].

**Cluster analysis **was performed using similarity coefficient based on bands that showed differences among isolates. Bionumerics software v 3 (Applied Maths, Belgium) was used to create UPGMA dendrogram.

## Results

### AFLP analysis

**Figure 1:**
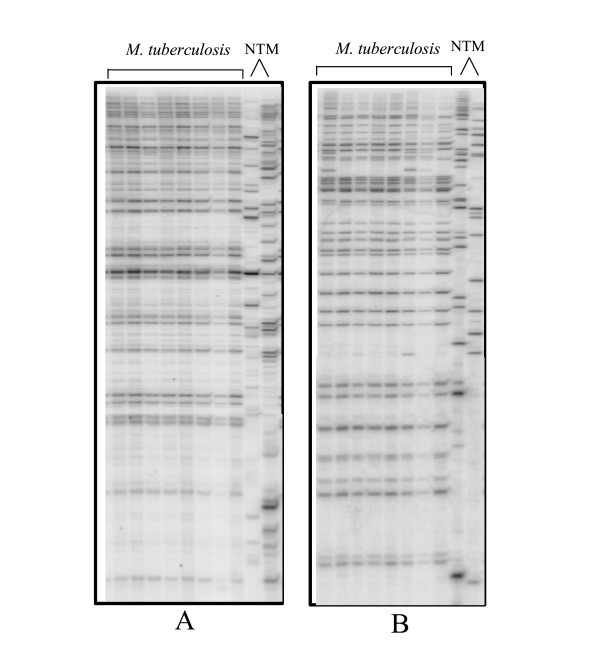
AFLP profiles generated by primer combinations EO/MT (panel A) and EG/MC (panel B). Lanes1-8: clinical isolates of *M. tuberculosis*, lanes 9&10: non tuberculous mycobacteria. The non-tuberculous mycobacteria have a very different profile from each other as well as from *M. tuberculosis *isolates. The primer pair EG/MC (panel B) shows fewer bands and more differences between the isolates as compared to EO/MT primer pair (panel A)

**Figure 2:**
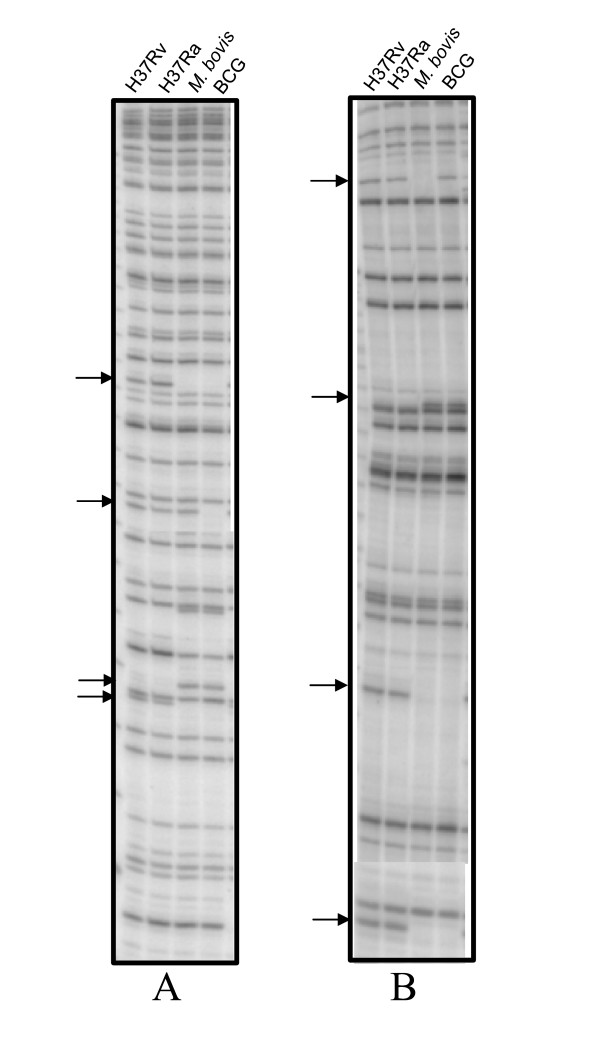
AFLP profiles showing differences (arrows) between *M. tuberculosis *H37Rv (lane1), H37Ra (lane 2), *M. bovis *(lane 3) and BCG (lane 4). The profiles shown are for EO/MC (panel A) and EO/MT (panel B) primer combinations.

The initial AFLP analysis of twenty five clinical isolates of *M. tuberculosis *using ten primer combinations showed that, combinations having a single selective primer yielded 40–50 bands, while any two selective primers, when combined, yielded only 15–35 bands. A band, if either absent or present in 10–90% of the isolates tested was considered to be polymorphic for the clinical isolates. Accordingly, there was relatively low polymorphism within the species (an average 4.27 % of the total number of bands/primer pair was polymorphic). But for two isolates of non-tuberculous mycobacteria, the AFLP profiles for all the primer combinations were highly different from those of *M. tuberculosis *(Figure 1). It was observed that the use of a selective primer pair increased the number of polymorphic bands while reducing the total number of bands obtained, whereas nonselective primer pairs had the opposite effect. Therefore, we analysed fifty clinical isolates of *M. tuberculosis *along with the laboratory strains of *M. tuberculosis, M. bovis *and BCG using four primer pairs, with each pair having one selective and one non-selective primer (EO/MT, EO/MG, EO/MA and EO/MC). Among these primer combinations, EO/MC combination showed maximum polymorphism within the *M. tuberculosis *species. The AFLP profiles for isolates of *M. bovis *and BCG did not vary much from those of *M. tuberculosis*, although there were a few bands that may be species-specific. In addition, there were a few differences between *M. bovis *and the BCG vaccine strain. Figure 2 shows the AFLP profiles for two primer combinations (EO/MC and EO/MT) that showed strain-specific differences.

### Comparison of AFLP with IS*6110*- RFLP

**Figure 3:**
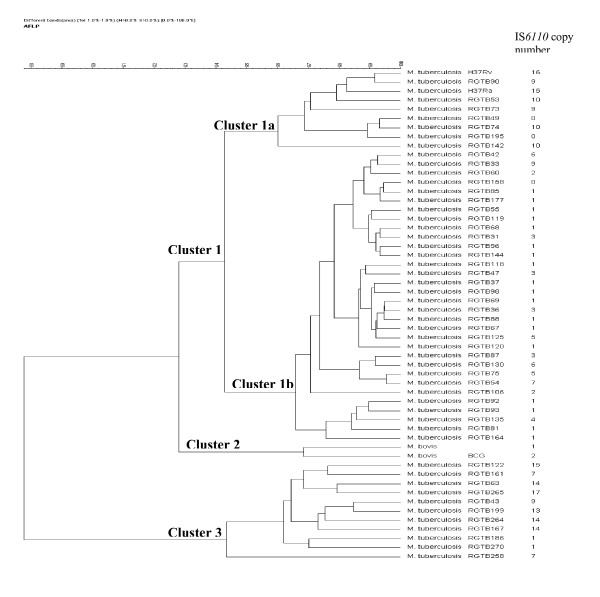
Dendrogram based on the AFLP profile for EO/MC primer pair showing clustering of clinical isolates of *M. tuberculosis *and laboratory strains of *M. tuberculosis*, *M. bovis *and BCG. IS*6110 *copy numbers of various isolates/strains are indicated.

Since we obtained maximum differences using the EO/MC primer pair, we used the AFLP profile obtained with this primer pair to construct a dendrogram (Figure 3) and compared this with the IS*6110 *fingerprinting data. Seventeen out of fifty isolates tested had IS*6110 *copy numbers ranging from 6 to 17. The remaining 33 were 'low copy' (0–5 copies) isolates, out of which 21 isolates had one, and three had no copy at all. This was in line with our earlier report on the high incidence of 'low copy isolates' in Kerala [3]. Cluster analysis showed three major clusters with the lowest similarity between them being 88%. The largest cluster (Cluster1) contained *M. tuberculosis *H37Rv, H37Ra and most of the clinical isolates. The subcluster 1a contained H37Rv, H37Ra and 7 clinical isolates, of which two were 'no copy' and five had 9 or 10 copies. Similarity within this subcluster ranged from 96–99.3%. The subcluster 1b had 28 (85%) 'low copy' and four high copy isolates, with similarity ranging from 96.4 to 99.5%. Cluster 2 contained only *M bovis *and BCG which are known to be similar. Eight 'high copy' isolates (with copies varying from 7 to 17) along with two single copy isolates formed Cluster 3. This cluster showed least intra-cluster similarity with values ranging from 94.2–98.5%. Overall, clusters 1 and 2 had 92.8% similarity to each other with only 88% similarity to cluster 3. The other 3 primer pairs showed fewer polymorphic bands, showed very little differentiation among the different isolates and were not considered for numerical analysis (data not shown).

## Discussion

In the present study we have used conventional radioactive AFLP in order to evaluate its potential to differentiate the *M. tuberculosis *isolates of Kerala. This is an endemic region where we have reported a high incidence of *M. tuberculosis *strains with low numbers of IS*6110 *which makes it difficult to be type them [3]. We used this method instead of multiplex Fluorescent AFLP, as we were also interested in developing AFLP-derived markers for the characterization of the Kerala isolates. However, the data presented in this paper is restricted to AFLP profiles and their comparison to IS*6110 *copy number data for a set of 50 isolates, of which 33 were 'low copy' isolates. Initially, our results on twenty-five clinical isolates showed 0–3 polymorphic bands per primer pair while using ten primer combinations. Using *Eco*R I/*Mse *I enzyme combination, approximately 4% of the total number of bands/primer combination was polymorphic for *M. tuberculosis*. There have been earlier reports on AFLP analysis of *Mycobacterium tuberculosis*. A study using multiplex FAFLP employing *Eco*R I and *Mse *I enzymes and four primer combinations had shown that 28% of the total number of fragments obtained were polymorphic [11]. In contrast, another study using radioactive AFLP and a different enzyme combination (*Apa *I/*Taq *I), demonstrated poor discrimination within the *M. tuberculosis *complex [10]. The discrepancy between these studies could be due to the choice of restriction enzymes and the methodology. FAFLP involves multiplexing which is not feasible with the radioactive methodology. Therefore, we have used a combination of the above methodologies using *Eco*R I/*Mse *I enzyme combination and conventional radioactive AFLP. Even though one may screen other enzyme combinations to increase polymorphism, our results suggest that radioactive AFLP is not an easy method for strain differentiation in endemic regions. However, AFLP could clearly differentiate *M. tuberculosis *from isolates of non-tuberculous mycobacteria. In our study, even though the close relationship between members of *M. tuberculosis *complex is reflected in AFLP analysis, the rare differences could also be detected. We characterized a few bands that differentiated *M. tuberculosis *and *M. bovis/*BCG by sequencing and identified three fragments belonging to RD1, RD4 and RD5 (results not shown). It has been reported that RD1 is absent only in BCG, while RD4-RD10 are deleted in both *M. bovis *and BCG [15]. Our results therefore correlate well with this data.

Comparing AFLP profile with IS*6110 *typing data showed that in AFLP analysis isolates clustered irrespective of the IS*6110 *copy number. The IS*6110 *'low copy' isolates were not well differentiated as nearly 85% of the low copy isolates clustered together (Fig 3, Cluster 1b). Therefore, it could be speculated that the low copy strains of Kerala are not very diverse. But all strains with a single copy of IS may not be identical, as they fell into different clusters (both 1 and 3) in the AFLP analysis. The high copy isolates were relatively better separated than the low copy isolates.

## Conclusion

We conclude that conventional radioactive AFLP alone does not seem to be an efficient fingerprinting method for *M. tuberculosis*. The combined use of IS*6110*-RFLP and AFLP again can differentiate the 'high copy' isolates to some extent, but does not differentiate the 'low copy' isolates efficiently. However the technique provides inter-species differentiation within and outside the *M. tuberculosis *complex and therefore is useful in the development of species-specific markers. The latest addition to the fingerprinting methods for *M. tuberculosis*, MIRU-VNTR [16,17], is efficient and is being adopted by several labs and we are currently analyzing our isolates using MIRU fingerprinting. But different fingerprinting techniques cluster the strains differently, and therefore generally appear to work independently of each other. Therefore, a unified fingerprinting system that can neatly pigeonhole all different strains is yet to be developed.

## Competing interests

The author(s) declare that they have no competing interests.

## Authors' contributions

MK carried out the AFLP analysis, cluster analysis and drafted the manuscrift

IR and BVJ carried out the IS*6110*-RFLP analysis

ML has helped in genomic DNA isolation and PCR

AK helped in drafting the manuscript and did the critical review

SM conceived of the study, and participated in its design and coordination and helped to draft the manuscript

## Pre-publication history

The pre-publication history for this paper can be accessed here:


